# Editorial: Health effects and pathophysiological mechanisms of one-carbon metabolism nutrients intake throughout the life cycle

**DOI:** 10.3389/fnut.2023.1269038

**Published:** 2023-08-22

**Authors:** Jufen Liu, Yunping Lei, Lei Wang, Chao Guo

**Affiliations:** ^1^Institute of Reproductive and Child Health, National Health Commission Key Laboratory of Reproductive Health, Peking University, Beijing, China; ^2^Department of Epidemiology and Biostatistics, School of Public Health, Peking University, Beijing, China; ^3^Department of Molecular and Cellular Biology, Baylor College of Medicine, Center for Precision Environmental Health, Houston, TX, United States; ^4^Department of Nutrition and Food Hygiene, School of Public Health, Capital Medical University, Beijing, China; ^5^Institute of Population Research, Peking University, Beijing, China

**Keywords:** prenatal nutrition, dietary supplementation, one-carbon metabolism nutrients, life cycle, pathophysiological mechanisms

The early-life nutritional environment, especially periconceptional nutrition, plays a critical role in shaping adverse health outcomes. These can encompass congenital malformations, development disorders, as well as later-life cardiovascular, cerebrovascular, and metabolic diseases. Folic acid supplementation before pregnancy has demonstrated its ability to prevent neural tube defects (NTDs) and other anomalies since the early 1990's (1–4), albeit with dosage variations across different populations. Despite extensive global public health efforts and increased awareness of the importance of supplementation, concerns about potential overdose risks persist (5). Recently the United States Preventive Services Task Force (USPSTF) reaffirms its 2017 recommendation (6) and gives the task force's highest A recommendation that all individuals who plan to or potentially could become pregnant take a daily supplement containing 0.4–0.8 mg folic acid, for the high certainty of substantial net benefit (7). Simultaneously, mounting evidence suggests that augmenting folic acid fortification could lead to even more significant reductions in associated risks (8). Consequently, comprehending the profound impact of nutrients during the inception of life and their enduring effects on the progression of multifactorial chronic complex diseases becomes imperative for advancing the realm of public health nutrition.

This Research Topic aims to bridge the gap in our knowledge about the health effects of early life nutrition status and dietary supplementation throughout an individual's life. The study focuses on various aspects, such as the epidemiology of folic acid supplements, potential genetic mechanisms, and a comprehensive overview of homocysteine in relation to health and disease. By presenting these findings, we hope to contribute significantly to promoting advancements in public health nutrition and wellbeing.

Periconceptional insufficiency of one-carbon metabolism nutrients, such as folate, vitamin B12, and other vitamins, increased the risk of neural tube defects and various adverse health outcomes. Yang et al. conducted a study to access the compliance of pregnant women in rural areas of Northwestern China regarding free folic acid supplements provided by the government and explored the role of related knowledge. Despite the government's efforts to distribute free folic acid, the actual usage among rural pregnant women in China remains suboptimal. This study investigated whether and how relevant knowledge affects pregnant women's ability to access and utilize the government-provided folic acid. The low rate of picking up and intake of free folic acid supplements was found to be primarily influenced by lack of policy awareness, and the timing and intention of pregnancy also affected folic acid intake. In addition, the author also found that advanced knowledge of nutrition and health was associated with a longer duration of folic acid intake but did not significant affect the uptake of folic acid before pregnancy. Therefore, to further reduce the risk of neural tube defects and other congenital malformations, the government needs to intensify policy promotion and ensure that rural women receive free folic acid supplements along with appropriate pre-pregnancy health and counseling services. The results of this study hold great significance for decision-makers in China and other developing countries to optimize the policy implementation.

Zhang et al. delved the health effect of homocysteine (Hcy), a critical intermediate product in the one-carbon metabolism methionine cycle, and explored the pathophysiological mechanisms through which the metabolites could impact health. Previous research has shown that abnormal Hcy metabolism is linked to B vitamin status, and correlates with cardio- and cerebrovascular disease, dementia, renal disease, thyroid disease, and pregnancy complications. Hcy is an intermediate product during the methionine cycle, and key enzymes such as Methylenetetrahydrofolate reductase (MTHFR), methionine synthase reductase (MTRR) and methionine synthase (MTR), play crucial roles in its metabolism, including participation in the transsulfuration pathway and the folate cycle. Specifically, the single nucleotide polymorphism (SNP) of the MTHFR gene (rs1801133, C667T) is associated with decreased enzyme activity, leading to increased plasma Hcy levels.

The objective of the study was to investigate the relationship between Hcy metabolism genes, Hcy levels, and early-onset post-stroke depression (PSD), providing novel insights into the pathogenesis of PSD. The results revealed that the PSD group had higher Hcy level than the non-PSD group. Additionally, the study found that the effect of MTHFR rs1801133 AA genotype on PSD was mediated by Hcy. In conclusion, this study suggested that Hcy levels and rs1801133 AA genotype, A allele, and haplotype GTAG (rs11559040-rs1801131-rs1801133-rs2066462) are associated with the an increased risk of early-onset PSD. These findings shed new light on the mechanisms underlying PSD and propose reducing Hcy concentration as a potential therapeutic target for prevention and intervention in PSD.

Finally, McCaddon and Miller provided a comprehensive review of Hcy. They traced the discovery, clinical significance, and its close relationship with one-carbon nutrients, folate, and vitamin B12 metabolism. The review explores its historical association with various diseases, including neural tube defects, cardio- and cerebrovascular disease and recently, dementia and Alzheimer's Disease. Additionally, it delves into recent controversies and considers potential future research directions, offering readers a general overview of homocysteine's role in health and disease.

The authors introduce two principal pathways of homocysteine metabolism: remethylation and transsulfuration, which play a closely coordinated role and are influenced by factors such as changes in dietary methionine intake, intracellular concentrations of S-adenosylmethionine (SAM), levels of dietary folate or vitamin B12, as well as the activity of critical enzymes like methylenetetrahydrofolate reductase (MTHFR), methionine synthase, and those involved in the synthesis of methylcobalamin. Impaired homocysteine metabolism can lead to various related disorders, including hyperhomocysteinemia, inborn errors of metabolism, cardio- and cerebrovascular disease, age-related cognitive impairment and dementia, as well as neural tube defects and other pregnancy outcomes. These findings represent significant progress in current research.

Firstly, hyperhomocysteinemia (HHcy) and homocystinuria are primarily associated with impairment in the remethylation and transsulfuration pathways or disruptions in their coordination. However, the effectiveness of lowering homocysteine with B vitamin supplements depends on individual baseline characteristics, requiring careful considerations to guide clinicians and patients in determining whether B vitamin supplementation could be beneficial for specific cases. As for age-related cognitive impairment and dementia, the biological mechanisms by which homocysteine-lowering with B vitamins might prevent cognitive decline and dementia have not yet been fully understood. Furthermore, homocysteine toxicity may be a contributing factor in the pathogenesis of neural tube defects or other pregnancy outcomes.

Homocysteine is at the intersection of folate and vitamin B12 metabolism, and the biological and clinical interaction between these two vitamins has been a subject of considerable interest for decades. Yet, there remain unresolved issues, such as the putative low B12/high folate interaction, associations between elevated serum levels of B12 and various outcomes, and the link between Alzheimer's disease (AD) and homocysteine. These areas continue to be subjects of ongoing exploration and require further study to gain comprehensive insights.

Advancements in diagnostics, personalized nutrition, epigenetics, and patient engagement are expected to play significant roles in unraveling the complexities of homocysteine's impact on health and disease. Homocysteine continues to be a promising biomarker and potential target for preventive interventions in the future.

Recommendation for periconceptional folic acid supplementation may be longstanding, but its implications remain fresh and vital. It stands as a crucial measure to safeguard the health of expectant mothers and avert birth defects, fostering not only the welfare of the individual but also contributing to the betterment of families, communities, and society as a whole (9).

## Author contributions

JL: Conceptualization, Writing—original draft, Writing—review and editing. YL: Writing—original draft, Writing—review and editing. LW: Writing—review and editing. CG: Writing—review and editing.
